# From Excision to Immunity: The Full Spectrum of Modern Melanoma Treatments

**DOI:** 10.3390/cancers18132043

**Published:** 2026-06-24

**Authors:** Vimal Murugesan, Thusanth Thuraisingam, Danuta Radzioch

**Affiliations:** 1 Department of Experimental Medicine, McGill University, Montreal, QC H4A 3J1, Canada; 2 Research Institute of the McGill University Health Centre, Montreal, QC H4A 3J1, Canada; 3 Division of Dermatology, Department of Medicine, McGill University, Montreal, QC H4A 3J1, Canada; 4 Department of Human Genetics, McGill University, Montreal, QC H4A 3J1, Canada

**Keywords:** cutaneous melanoma, surgery, chemotherapy, immunotherapy, targeted therapy

## Abstract

Cutaneous Melanoma is a biologically heterogeneous malignancy. Although recent therapeutic advances have improved survival, durable remissions remain elusive for many patients. Surgical excision with stage-appropriate margins and selective nodal staging remains the cornerstone of curative-intent management. In contrast, conventional cytotoxic chemotherapy now plays a limited, largely palliative role given its modest efficacy and substantial toxicity. Targeted therapy with BRAF/MEK inhibitors has improved outcomes in patients with *BRAF* V600-mutant melanoma, resulting in rapid tumor regression and meaningful survival benefits. However, long-term disease control is frequently compromised by adaptive resistance, commonly driven by MAPK pathway reactivation or compensatory PI3K/AKT signaling. In parallel, immune checkpoint inhibitors targeting PD-1, CTLA-4, and emerging pathways have reshaped treatment across disease stages, enabling deep and sometimes durable responses. Despite this progress, primary and acquired resistance, as well as acute and chronic immune-related toxicities, continue to pose significant clinical challenges. Current therapeutic strategies focus on rational combinations of targeted therapy, checkpoint blockade, IL-2-based approaches, oncolytic viruses, and adoptive cell therapies such as tumor-infiltrating lymphocytes to enhance response depth and durability. However, these intensified regimens carry increased toxicity risks, highlighting the need for improved patient selection and monitoring. Overall, emerging evidence supports a paradigm shift toward optimized treatment sequencing, response-adapted surgical strategies, and biomarker-guided personalization to maximize clinical benefit while minimizing toxicity.

## Simple Summary

Melanoma is a skin cancer that has seen dramatic treatment advances over the past two decades. Surgery is the foundation of care for early-stage disease, but for advanced melanoma, two breakthroughs have transformed outcomes. First, drugs that precisely target a common gene mutation can shrink tumors rapidly. Second, immunotherapy—treatments that activate the body’s own immune system to fight cancer—can produce long-lasting remissions in patients who previously had almost no options. Newer approaches, including infusing a patient’s own tumor-fighting immune cells (TIL therapy) and virus-based treatments, are expanding what is possible. The central challenge now is selecting the right treatment, in the right sequence, for each individual patient.

## 1. Introduction

Cutaneous melanoma is among the most aggressive forms of skin cancer, characterized by a high propensity for regional and distant metastasis and by a remarkable capacity to evade both endogenous immune surveillance and systemic therapeutic pressure. Surgical excision has been the only curative modality for localized disease. Beyond the primary tumor, however, outcomes were for decades determined by a disease that responded poorly to cytotoxic chemotherapy, with dacarbazine as the longtime standard agent producing objective responses in fewer than one in five patients and median overall survival of six to nine months in the metastatic setting [1].

The landscape was fundamentally altered by two near-simultaneous discoveries: the identification of activating *BRAF* mutations in approximately 50% of cutaneous melanomas, and the demonstration that blocking immune checkpoint molecules could reinvigorate durable anti-tumor immunity. Vemurafenib, the first BRAF inhibitor approved for *BRAF* V600E-mutant melanoma, and ipilimumab, the first anti-CTLA-4 antibody, both achieved regulatory approval in 2011, inaugurating an era in which advanced melanoma was transformed from a rapidly fatal disease into one in which long-term survival is achievable for a meaningful subset of patients [2,3].

The subsequent decade brought further depth to both approaches. Dual BRAF/MEK inhibition superseded BRAF inhibitor monotherapy by reduced acquired resistance and cutaneous toxicity. Anti-PD-1 agents demonstrated superior single-agent activity compared with ipilimumab, and their combination with anti-CTLA-4 therapy produced synergistic but toxicity-laden responses. Novel checkpoint targets, most recently LAG-3, antagonized by relatlimab have expanded the range of dual checkpoint inhibition with a more favorable safety profile. Tumor-infiltrating lymphocyte therapy has opened a new frontier in adoptive cellular immunotherapy for checkpoint-refractory disease and neoadjuvant immunotherapy with response-directed surgical de-escalation has begun to redefine the perioperative management of resectable stage III disease.

This narrative review focuses on systemic and perioperative therapeutic strategies for adult patients with cutaneous melanoma. A literature search was conducted in PubMed/MEDLINE, Embase, and Web of Science for studies published between January 2010 and March 2026, using combinations of keywords including “cutaneous melanoma,” “immunotherapy,” “checkpoint inhibitors,” “BRAF/MEK inhibitors,” and “adoptive cell therapy.” Relevant clinical trials, meta-analyses, and high-impact prospective and retrospective studies were prioritized, and additional articles were identified through screening.

### International Guideline Disagreements: NCCN vs. ESMO vs. EADO/EDF vs. ANZMTAG

Significant discrepancies exist across international melanoma guidelines. The most prominent disagreement concerns sentinel lymph node biopsy (SLNB) thresholds: NCCN recommends SLNB for melanomas ≥ 0.8 mm Breslow thickness (or ≥0.75 mm in some versions) and considers it optional for thin melanomas with high-risk features, whereas ESMO recommends SLNB for pT2a (>1.0 mm) and suggests discussing it for pT1b (0.8–1.0 mm), taking a more conservative approach. Adjuvant therapy for stage IIB–IIC represents another key divergence: NCCN strongly recommends adjuvant anti-PD-1 therapy (pembrolizumab or nivolumab) for these high-risk stage II patients, while ESMO frames it as optional after detailed risk-benefit discussion, reflecting differences and pointing out how each panel weighs recurrence benefit versus toxicity [4].

Neoadjuvant therapy acceptance varies-NCCN Version 2.2024 formally incorporated neoadjuvant pembrolizumab based on SWOG S1801 data, while ESMO notes it is not EMA/FDA-approved but can be considered in trials. The EADO/EDF European dermatology-guidelines emphasize dermatologic surveillance and take a more conservative stance on systemic therapy initiation compared to NCCN’s oncology-driven aggressiveness. ANZMTAG (Australia/New Zealand) uniquely incorporates local epidemiology with higher incidence rates and often recommends more intensive surveillance intervals than NCCN or ESMO for high-risk patients, and follow-up different imaging schedules. Additionally, complete lymph node dissection (CLND) after positive SLNB is strongly recommended against by ESMO (based on MSLT-II and DeCOG-SLT data), while NCCN acknowledges this but still lists it as a consideration in select cases. These differences reflect varying interpretations of evidence quality, regional drug approval status (EMA vs. FDA), and risk tolerance [5,6,7].

## 2. Surgical Management of Melanoma

### 2.1. Melanoma In Situ and Lentigo Maligna: Surgical Margins and Management

Melanoma in situ (MIS) and its most common subtype, lentigo maligna (LM), represent a biologically and surgically distinct entity from invasive melanoma, characterized by unique margin challenges that standard wide local excision (WLE) guidelines often fail to capture. Although current NCCN guidelines recommend 0.5–1.0 cm excision margins for MIS; these margins are frequently insufficient for LM, which has a well-recognized tendency for extensive subclinical spread along the dermo-epidermal junction beyond the clinically apparent border [4,8].

Histopathological studies consistently show that standard 5 mm excision margins clear fewer than half of LM lesions, underscoring the inadequacy of conventional recommendations for this subtype. Subclinical extension of atypical melanocytes can exceed 0.5–2.0 cm beyond the clinically apparent border, an issue particularly pronounced on chronically sun-damaged facial skin, where background solar lentigines and lichenoid inflammation create interpretive challenges for standard bread-loaf histopathological assessment, and where field cancerization further complicates margin evaluation. As a result, achieving histologically clear margins often requires wider excisions than initially anticipated, with important implications for reconstructive planning and functional outcomes, particularly in anatomically sensitive areas such as the nose, eyelids, and ears [9,10].

### 2.2. Mohs Micrographic Surgery and Staged Excision for Melanoma In Situ

Mohs micrographic surgery (MMS) and related staged margin-controlled excision techniques have become preferred approaches for MIS and LM in anatomically sensitive or cosmetically critical areas. These methods offer the dual advantage of complete peripheral and deep margin assessment while maximizing tissue preservation.

Unlike conventional bread-loaf sectioning, which samples only a small fraction of the surgical margin MMS uses horizontal frozen sections that evaluate nearly 100% of the peripheral and deep margin. This approach enables real-time margin control and reduces the risk of local recurrence due to undetected subclinical extension [11]. Multiple retrospective studies and a 2023 systematic review and meta-analysis demonstrate that MMS achieves lower local recurrence rates for LM and MIS. MMS with immunohistochemistry achieves recurrence rates of 0–3% at five or more years of follow-up, compared with recurrence rates of 6–20% following WLE using the guideline-recommended 5 mm surgical margins for MIS. In addition, MMS allows for smaller initial excisions and staged reconstruction, facilitating preservation of critical anatomical structures [10,12]. Several major guidelines now recognize MMS as an acceptable—and, in select cases, preferred—approach for MIS. The 2024 NCCN guidelines list MMS as an option for MIS in anatomically sensitive areas, and the American College of Mohs Surgery endorses its use for LM and MIS when margin control and tissue conservation are priorities.

A related technique, staged excision with permanent sections (often referred to as “slow Mohs” or the square procedure), provides margin-control advantages using formalin-fixed paraffin-embedded sections interpreted by a dermatopathologist [4]. This approach offers superior cytological detail relative to frozen sections while still enabling complete peripheral and deep margin assessment making it particularly valuable in challenging anatomical sites where frozen-section artifact on sun-damaged skin may limit interpretability [13]. Despite these advantages, MMS for melanoma is more resource-intensive than standard WLE, requires centers with specialized expertise in melanocytic frozen section interpretation, and is not uniformly reimbursed across health systems. These factors continue to restrain its broader adoption outside of high-volume academic or specialty centers.

### 2.3. Re-Excision Following Positive or Close Surgical Margins

Positive or close surgical margins after initial WLE are a common clinical scenario that requires careful, individualized management. A positive deep margin—typically defined as melanoma cells present at or within 1 mm of the deep surgical edge—carries a higher risk of local recurrence and often indicates residual disease that warrants re-excision. In contrast, a positive peripheral margin may reflect either remaining primary tumor or, in the case of MIS with subclinical radial spread, a need for broader margin clearance [4].

Current guidelines recommend re-excision to achieve clear margins whenever technically feasible, with the extent of additional surgery guided by the original tumor’s Breslow thickness, anatomical location, and whether the involved margin contains in situ or invasive melanoma [7]. In anatomically constrained areas such as the periorbital region, nose, or digits—where guideline-recommended margins would result in substantial functional or cosmetic morbidity, multidisciplinary input from dermatologic surgery, plastic surgery, and oncology is essential to balance oncologic adequacy with quality of life. In these settings, staged margin-controlled techniques, including MMS or slow Mohs, may provide a tissue-sparing alternative capable of achieving complete margin clearance without the morbidity associated with wide re-excision. For invasive melanoma with a focally positive deep margin in which re-excision is not feasible, adjuvant radiation therapy may be considered as part of a multimodal strategy, although evidence supporting its routine use in this context is limited [14].

Histopathological evaluation of surgical margins is essential. Pathologists assess the microscopic linear distance between melanoma cells and the inked surgical margin, but interpretation must account for tissue shrinkage: surgical specimens typically contract by 15–25% during processing, which can meaningfully affect measured margin width. Post-operative complications of WLE include infection (reported in up to 5–10% of cases), wound-healing difficulties, seroma formation, bleeding, and scarring. Larger excisions more frequently necessitate reconstructive procedures—such as skin grafts or local flaps, particularly for lesions on the trunk and extremities. Although these approaches increase surgical morbidity and recovery time, they may be appropriate and justified in carefully selected patients [15,16].

### 2.4. Sentinel Lymph Node Biopsy

SLNB plays a central role in melanoma staging and prognostication. Current ASCO/SSO guidelines state that SLNB is not routinely recommended for T1a melanomas but may be considered for disease with ulceration [17]. SLNB is a minimally invasive surgical staging procedure that uses lymphatic mapping with radiotracer and/or vital blue dye to identify the sentinel lymph node(s)-the first draining node(s) most likely to harbor metastasis. SLNB provides important prognostic information and can detect occult nodal micro metastases that would otherwise remain undetected [18,19].

Current guidelines from AIOM, ESMO, and NCCN uniformly endorse SLNB as the standard staging procedure for intermediate and high-risk primary melanomas, typically those with Breslow thickness > 1.0 mm, and recommend that it be discussed for selected T1b lesions (0.8–1.0 mm or <0.8 mm with ulceration or other adverse features). Compared with clinical nodal assessment alone, SLNB offers superior staging precision, enabling up staging of patients with occult nodal involvement and identifying those who may benefit from adjuvant systemic therapy [4,15,16,17]. However, the therapeutic value of SLNB remains an area of active debate.

The role of SLNB in guiding completion lymph node dissection (CLND) has evolved with recent evidence indicating that not all sentinel node-positive patients benefit from immediate CLND. The MSLT-II trial (Multicenter Selective Lymphadenectomy Trial II) demonstrated that ultrasound surveillance provides melanoma-specific survival comparable to immediate CLND, despite a higher rate of regional nodal recurrence with observation. These findings were corroborated by the DeCOG-SLT (Dermatologic Cooperative Oncology Group Sentinel Lymph Node Trial) trial, a German multicenter randomized study, which similarly showed no significant differences in distant metastasis-free survival or melanoma-specific survival between immediate CLND and nodal observation. The DeCOG-SLT included patients with minimal nodal tumor burden (maximum metastasis diameter ≤ 1 mm), further supporting the conclusion that routine immediate CLND does not confer a survival advantage, particularly in this subgroup [20,21].

Although SLNB provides important prognostic information—particularly for patients with intermediate thickness melanomas (approximately 1–4 mm)—randomized trials conducted prior to the immunotherapy era did not demonstrate a clear overall survival benefit of an SLNB based strategy compared with nodal observation. The MSLT II trial showed that ultrasound surveillance of sentinel node positive patients yields melanoma specific survival comparable to immediate CLND, although regional nodal recurrence rates were higher with observation [22,23,24].

The findings of MSLT II were independently corroborated by the DeCOG SLT trial, a German multicenter randomized controlled study that likewise demonstrated no significant difference in distant metastasis free survival or melanoma specific survival between immediate CLND and nodal observation with ultrasound surveillance in sentinel node positive patients. Notably, DeCOG SLT enrolled patients with minimal nodal tumor burden (maximum metastasis diameter ≤ 1 mm), further supporting the conclusion that routine immediate CLND does not provide a survival advantage in this subgroup [18,20].

For patients with high risk T3b–T4 melanomas, the value of SLNB is increasingly being reconsidered in the era of effective adjuvant immunotherapy, because many of these individuals are now candidates for systemic treatment based on primary tumor characteristics alone. The 2024 NCCN guidelines acknowledge this evolving landscape by emphasizing shared decision making around SLNB in high-risk stage II melanoma (IIB–IIC), rather than mandating the procedure in all cases, but this does not establish broad omission as standard practice [4]. In selected patients-particularly when adjuvant therapy is already planned or when patient preferences and surgical morbidity are important considerations, the omission of SLNB may be discussed, but it is not currently established as a broad standard approach. In selected patients-particularly when adjuvant therapy is already planned, patient preferences favor avoiding additional surgery, or the potential morbidity of SLNB is a concern, omission of SLNB may be considered reasonable. However, SLNB remains the standard method for accurate pathologic staging, and its role continues to be important for prognostication and treatment planning [25,26].

### 2.5. Lymphadenectomy and Regional Nodal Management

Therapeutic lymph node dissection (TLND) is performed to manage clinically evident regional lymph node metastases identified through imaging or clinical examination. Complete lymph node dissection (CLND) still is the standard surgical approach for confirmed nodal involvement, though its extent has been refined with contemporary evidence [27].

The role of completion lymph node dissection (CLND) following a positive SLNB has been reevaluated considering landmark randomized trials and real-world experience. Historically, CLND was routinely performed for all SLNB-positive patients, based on the assumption that the sentinel node reflected the status of the entire nodal basin. Contemporary evidence, however, supports a more selective, individualized approach, with many SLNB-positive patients now managed with nodal observation and ultrasound surveillance rather than immediate CLND. In parallel, the shift toward response adapted and de-escalation strategies particularly with the emergence of neoadjuvant immunotherapy for clinically node positive disease has further refined the role of therapeutic lymph node dissection within the modern melanoma treatment paradigm [28].

Post-operative complications vary considerably depending on the extent of lymph node dissection and whether reconstruction is required. Infection occurs in approximately 2–10% of cases, influenced by wound size, anatomical location, and patient-specific factors. Lymphedema is the most common long-term morbidity after lymph node dissection, developing in approximately 10–40% of patients depending on nodal basin (inguinal > axillary > cervical) and extent of surgery, and it can significantly impair quality of life. Seroma formation occurs in 5–15% of patients—particularly after extensive dissections—and may require repeated drainage. Additionally, nerve injury during nodal dissection can result in permanent sensory deficits or, less commonly, motor dysfunction, depending on the nerves involved. The cumulative morbidity associated with extensive surgical procedures must be weighed against the therapeutic benefit, particularly highly effective systemic therapies [28,29].

## 3. Chemotherapy in Melanoma

Chemotherapy historically served as the cornerstone of melanoma treatment, but its role has diminished with the advent of targeted therapies and immunotherapy. Dacarbazine (DTIC), an alkylating agent approved in 1975, was the standard for decades, producing response rates of approximately 20% and median overall survival of 6–9 months. As an imidazole-carboxamide prodrug, DTIC undergoes hepatic activation to generate DNA-alkylating metabolites that induce cross-linking and tumor cell death [1,30]. Temozolomide (TMZ), an oral analog with improved pharmacokinetics and central nervous system penetration, offered greater convenience but failed to demonstrate survival advantages over DTIC, with similar response rates of 20–25% [1,31].

Attempts to improve outcomes through combination regimens yielded limited success. Multi-agent protocols such as the Dartmouth regimen and cisplatin-based combinations increased toxicity without meaningful survival benefit. Similarly, “Biochemotherapy” approaches combining chemotherapy with cytokines (interferon-α or interleukin-2) improved response rates but did not translate into durable survival gains and were associated with substantial toxicity [1].

In contemporary practice, chemotherapy is largely restricted to a palliative role in patients who are ineligible for, or have progressed after, immunotherapy and targeted therapies. It may be considered in select cases, including rapidly progressive visceral disease requiring cytoreduction or contraindications to immunotherapy. However, its clinical utility is limited by modest efficacy, short-lived responses (typically 3–6 months), and a well-established toxicity profile including myelosuppression, gastrointestinal effects, and infection risk [1,31,32].

These limitations are driven in large part by intrinsic and acquired resistance mechanisms. Melanoma exhibits relative resistance to alkylating agents despite a high mutational burden, mediated by enhanced DNA repair capacity, mismatch-repair deficiencies, and increased expression of O6-methylguanine-DNA methyltransferase (MGMT). Additional contributors include drug efflux transporter upregulation and impaired apoptotic signaling, including alterations in p53 pathways and mitochondrial dynamics [31].

Efforts to overcome resistance have explored combination strategies with targeted approaches. Preclinical studies in A375 melanoma cells show that *BRAF* suppression enhances DTIC or TMZ-induced cytotoxicity, partly through increased caspase-3 activation and apoptosis. While these findings suggest potential synergy, clinical translation is limited, reinforcing the marginal role of chemotherapy in the current therapeutic landscape [1,31].

## 4. Targeted Therapies: BRAF and MEK Inhibition

### 4.1. BRAF Mutation Biology and Inhibitor Development

The *BRAF* V600E mutation occurs in approximately 50% of cutaneous melanomas, making it the most common activating mutation in melanoma. This mutation leads to constitutive activation of the *BRAF* serine/threonine kinase, driving persistent signaling through the mitogen-activated protein kinase (*MAPK*) pathway independent of upstream receptor activation. As a clear oncogenic driver, *BRAF* V600E provides a compelling therapeutic target. First-generation BRAF inhibitors—mostly vemurafenib and dabrafenib—demonstrated striking single-agent activity, achieving response rates of 40–60% in *BRAF*-mutant metastatic melanoma and producing dramatic improvements in progression-free survival compared with chemotherapy [33,34].

However, BRAF inhibitor monotherapy revealed a critical paradox: in cells lacking *BRAF* mutations—including wild-type melanoma cells and various immune and stromal populations—these agents can activate rather than inhibit the MAPK pathway. This paradoxical activation arises from relief of feedback inhibition and transactivation of other RAF kinases, particularly in the setting of upstream RTK signaling. Clinically, this manifests as BRAF inhibitor-associated cutaneous proliferations, including keratoacanthomas and other benign skin eruptions, occurring in approximately 25–30% of treated patients, and reflecting visible consequences of unintended MAPK pathway activation [33]. The solution to BRAF inhibitor-associated toxicity, as well as the need for improved therapeutic efficacy, emerged with the addition of MEK inhibitors, which block the kinase immediately downstream of *BRAF* in the MAPK cascade. Combination BRAF/MEK therapy—using regimens such as dabrafenib plus trametinib, vemurafenib plus cobimetinib, or encorafenib plus binimetinib demonstrated clear superiority over BRAF-inhibitor monotherapy.

These combinations produced higher response rates of 60–70%, significantly prolonged progression-free survival, reduced cutaneous toxicities associated with paradoxical MAPK activation, and improved overall patient survival across multiple randomized controlled trials. As a result, dual BRAF/MEK inhibition now represents the standard of care for *BRAF*-mutant metastatic melanoma when targeted therapy is selected as first-line treatment [1,33,35,36,37,38]. Clinical development has also been limited by dose-limiting toxicities (including ocular, gastrointestinal, and cutaneous effects), modest and often short-lived responses, and evidence of pathway rebound or compensatory feedback when used as monotherapy. Combination of the ERK inhibitor ravoxertinib (GDC-0994) with a MEK inhibitor led to intolerable overlapping toxicities in advanced solid tumors, limiting feasibility [37,38].

Despite these limitations, preclinical data support the concept that vertical inhibition of the MAPK pathway—combining ERK inhibitors with BRAF and/or MEK inhibitors—can significantly enhance pathway suppression and overcome resistance, particularly in chronic treatment settings. Ongoing clinical trials are evaluating ERK inhibitors in rational combinations with BRAF/MEK inhibitors, CDK4/6 inhibitors, PI3K/AKT/mTOR inhibitors, or immunotherapy to improve durability and mitigate resistance. If these strategies succeed, ERK inhibitors could become a meaningful next-generation extension of MAPK-targeted therapy; at present, however, they remain an emerging rather than established component of standard care for *BRAF*-mutant melanoma [35,37].

### 4.2. Resistance Mechanisms to BRAF and MEK Inhibitors

Despite the initial dramatic response to MAPK inhibition, nearly all patients with *BRAF*-mutant melanoma eventually develop resistance to BRAF/MEK inhibition, with median progression-free survival of 10–12 months. Resistance mechanisms are diverse and multifactorial, involving both genetic alterations and epigenetic or transcriptomic reprogramming. Approximately 50% of resistant tumors harbor identifiable genetic drivers of resistance, while the remaining 50% progress through non-genomic mechanisms such as epigenetic remodeling and transcriptional adaptation [33,34].

Genetic resistance mechanisms frequently involve reactivation of the MAPK pathway through a range of alterations, including overexpression of upstream receptor tyrosine kinases such as MET and PDGF, activating *NRAS* mutations, amplification of the mutant *BRAF* allele, and expression of BRAF splicing variants. These BRAF splice variants—present in approximately 13–30% of BRAFi-resistant melanomas—encode truncated BRAF proteins that evade inhibitor binding and sustain MAPK signaling. Mutations in MEK1/2 also contribute to resistance, occurring in roughly 7–8% of cases; specific alterations (e.g., C121S, E203K, Q56P, K57E in MEK1; E207K, Q60P in MEK2) enable downstream ERK activation despite MEK inhibition, thereby maintaining proliferative signaling [33,34,39].

Epigenetic and transcriptomic mechanisms account for resistance in 40–50% of resistant tumors that lack identifiable genetic alterations [40]. These non-genomic adaptations include upregulation of alternative pathways such as PI3K/AKT, Wnt, as well as shifts in transcriptional programs—mostly MITF amplification, which drives metabolic rewiring toward OXPHOS and reduces dependence on MAPK signaling. Additional changes in immune-related gene expression further contribute to therapeutic escape, including loss of antigen-presentation components (B2M, HLA-A, HLA-B, TAP1) and reduced expression of genes involved in adaptive immunity, promoting immune evasion [40,41].

The tumor microenvironment also plays a pivotal role in therapeutic resistance. Stromal HGF secretion activates MET signaling and reengages the PI3K/AKT pathway, while macrophage infiltration and paracrine VEGF production promotes MAPK pathway activation in both macrophages and melanoma cells [34,42]. BRAFi/MEKi therapy profoundly reshapes the tumor microenvironment, generating both immune-stimulatory and immune-suppressive effects over time. Early during treatment, BRAF inhibition increases melanoma antigen expression (MART-1, TYRP1, gp100) enhances CD8+ T-cell infiltration, and reduces myeloid-derived suppressor cells, creating a more immunogenic milieu. As resistance emerges, however, the microenvironment shifts back toward immunosuppression, characterized by reduced antigen expression, increased T-cell exhaustion, and the upregulation of PD-L1. These changes highlight the role of immune escape in clinical resistance and provide strong rationale for combining BRAF/MEK inhibitors with immune checkpoint blockade [33].

## 5. Immunotherapy: Checkpoint Inhibitors and Beyond

### 5.1. Anti-PD-1 and Anti-CTLA-4 Monotherapy

The discovery that programmed cell death-1 (PD-1) and cytotoxic T-lymphocyte-associated antigen-4 (CTLA-4) function as critical immune checkpoints transformed the therapeutics landscape of melanoma. PD-1, an inhibitory receptor expressed on T cells and other immune cells, suppresses T-cell activity upon engagement with its ligands PD-L1 and PD-L2 on tumor and stromal cells. CTLA-4, also expressed on T cells, binds B7 molecules on antigen-presenting cells and attenuates T-cell activation at an earlier stage of the immune response, primarily within lymphoid organs where T-cell priming occurs [3].

Anti-PD-1 monotherapy using agents such as pembrolizumab or nivolumab achieves objective response rates of approximately 40–45% in patients with advanced melanoma, with median progression-free survival of 6–9 months and median overall survival exceeding 12–18 months in contemporary trials. About one-third of patients achieve durable five-year survival—a dramatic improvement over historical outcomes with chemotherapy or high-dose IL-2. In contrast, anti-CTLA-4 monotherapy with ipilimumab produces lower response rates of 15–20%, with median overall survival of 12–14 months [3,43,44].

**Figure 1:**
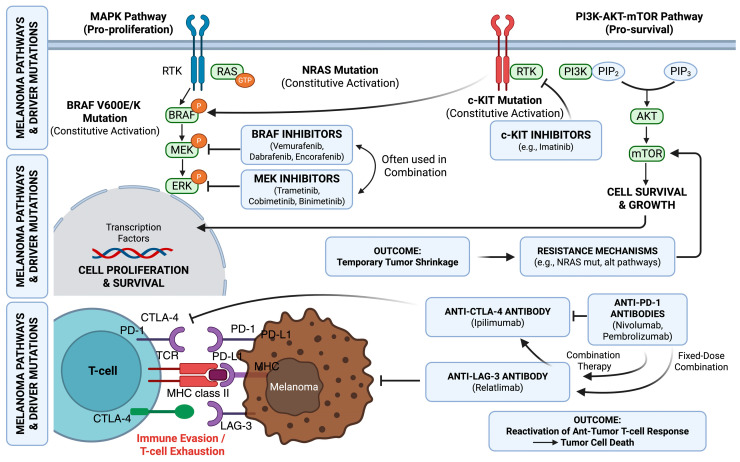
Schematic of key signaling pathways and therapeutic targets in melanoma. Created in https://BioRender.com. Schematic illustration of the MAPK and PI3K/AKT/mTOR signaling pathways activated by common melanoma driver mutations, including *BRAF* V600E/K, *NRAS*, and *KIT*. In the MAPK cascade, receptor tyrosine kinase (RTK) signaling activates RAS, BRAF, MEK, and ERK, promoting melanoma cell proliferation and survival. In parallel, RTK-driven PI3K activation leads to PIP2 conversion to PIP3, AKT activation, and downstream mTOR signaling, which supports cell survival and growth. Therapeutic interventions are indicated, including BRAF inhibitors (vemurafenib, dabrafenib, encorafenib), MEK inhibitors (trametinib, cobimetinib, binimetinib), and c-KIT inhibitors (e.g., imatinib). Immune checkpoint blockade with anti-PD-1, anti-PD-L1, and anti-CTLA-4 antibodies enhances T-cell anti-tumor activity by relieving inhibitory signaling and promoting antigen presentation and immune activation. Arrows indicate oncogenic signaling, whereas inhibitory symbols denote sites of pharmacologic intervention. Abbreviations: AKT, protein kinase B; BRAF, v-raf murine sarcoma viral oncogene homolog B1 protein; c-KIT/KIT, KIT proto-oncogene receptor tyrosine kinase; CTLA-4, cytotoxic T-lymphocyte-associated protein 4; ERK, extracellular signal-regulated kinase; MAPK, mitogen-activated protein kinase; MEK, mitogen-activated protein kinase; mTOR, mechanistic target of rapamycin; NRAS, neuroblastoma RAS viral oncogene homolog protein; PD-1, programmed cell death protein 1; PD-L1, programmed death-ligand 1; PI3K, phosphoinositide 3-kinase; PIP2, phosphatidylinositol 4,5-bisphosphate; PIP3, phosphatidylinositol 3,4,5-trisphosphate; RAS, rat sarcoma; RTK, receptor tyrosine kinase.

The mechanisms of action of these two agents differ in fundamental ways. Anti-PD-1 therapy relieves inhibitory signaling on pre-existing tumor-infiltrating CD8+ T cells by blocking the PD-1/PD-L1 interaction, thereby restoring effector T-cell function in tumors that are already inflamed. In contrast, anti-CTLA-4 therapy acts earlier in the immune response, broadening the T-cell repertoire by enhancing T-cell priming and reducing regulatory T cell (Treg)-mediated suppression, thus expanding the pool of tumor-reactive T cells capable of infiltrating tumors. These complementary mechanisms provide a strong rationale for combining checkpoint blockade therapies [3]. Figure 1 describes the key signaling pathways involved in Melanoma.

### 5.2. Combination Anti-CTLA-4 and Anti-PD-1 Checkpoint Blockade

The combination of anti-CTLA-4 (ipilimumab, 3 mg/kg) plus anti-PD-1 (nivolumab, 1 mg/kg) has demonstrated synergistic efficacy, achieving response rates of 55–58%, higher than those seen with either agent alone. In the long-term follow-up of the CheckMate 067 trial—now extending beyond 7.5 years—median melanoma-specific survival (excluding non-melanoma deaths) has not yet been reached in the combination arm, underscoring unprecedented durability of this regimen. However, the benefit comes with substantially increased toxicity: grade 3–4 treatment-related adverse events occurring in 55% of patients receiving combination therapy, compared to 16% for nivolumab monotherapy and 27% for ipilimumab alone [45].

The superior efficacy and substantial toxicity of combination immunotherapy make careful patient selection essential. In clinical practice, combination therapy is generally reserved for individuals with high-risk features—such as visceral metastases, elevated LDH, rapid disease progression, or brain metastases—who also have sufficient performance status to tolerate the increased risk of severe immune-related adverse events. To support this decision-making process, predictive models incorporating baseline clinical factors have been developed to identify patients who are likely to derive comparable benefit from anti-PD-1 monotherapy, allowing them to avoid the added toxicity of combination regimens [43].

### 5.3. Novel Checkpoint Inhibitors: LAG-3 and Beyond

Recent FDA approvals have further expanded the landscape of checkpoint inhibition in melanoma. Relatlimab, a novel LAG-3 inhibitor, was approved in combination with nivolumab (marketed together as Opdualag) following the RELATIVITY-047 trial (RELATIVITY trials are studies on relatlimab) which demonstrated a significant improvement in progression-free survival compared to nivolumab monotherapy (HR 0.75, *p* = 0.0055). This dual regimen achieved its clinical benefits with a more favorable safety profile than ipilimumab and nivolumab combination, showing lower rates of grade 3–4 adverse events (22%) while still enhancing clinical efficacy through improved progression-free survival. This represents a meaningful advance, broadening the population of patients who may benefit from dual checkpoint inhibition without the prohibitive toxicity associated with ipilimumab/nivolumab [43,46,47].

Additional immune-checkpoint targets continue to emerge as clinically relevant regulators of anti-tumor immunity. Fianlimab, a human monoclonal antibody against LAG-3, is currently being evaluated in combination with the anti-PD-1 antibody cemiplimab in a phase 3 clinical trial for both adult and adolescent patients with melanoma (NCT05352672) [48,49,50,51]. Like PD-1, inhibitory receptors such as TIM-3 and TIGIT are upregulated on exhausted T cells in the TME, and their blockade represents a promising strategy to reinvigorate anti-tumor immunity. As understanding of immune evasion pathways deepens, rational multi-checkpoint inhibition strategies are rapidly expanding across melanoma and other solid tumors [52].

### 5.4. Tumor-Infiltrating Lymphocyte (TIL) Therapy

Tumor-infiltrating lymphocyte (TIL) therapy has recently emerged as a clinically validated form of adoptive cellular immunotherapy for advanced melanoma, particularly in patients who have progressed on PD-1-based checkpoint blockade and, where applicable, BRAF/MEK inhibitors. In February 2024 (Table 1), the FDA granted accelerated approval to lifileucel (Amtagvi)—the first commercially manufactured autologous TIL product and the first cellular therapy approved for a solid tumor—for adults with unresectable or metastatic melanoma previously treated with PD-1 inhibitors and targeted therapy when indicated. Lifileucel is generated by harvesting T cells from a resected melanoma lesion, expanding these tumor-reactive lymphocytes ex vivo, and reinfusing them following non-myeloablative lymphodepleting chemotherapy and high-dose interleukin-2. This “one-and-done” infusion boosts the breadth and function of endogenous anti-tumor T cells. By leveraging a polyclonal, patient-specific T-cell repertoire directed against multiple melanoma neoantigens, TIL therapy offers a mechanistically distinct and complementary strategy to checkpoint inhibition, which primarily modulates existing T-cell activity in vivo [53,54,55,56,57].

Efficacy data from the pivotal C-144-01 trial and its long-term follow-up underpin lifileucel approval. In the most recent 5-year analysis (20 November 2024), the objective response rate (ORR) was 31.4% (complete response [CR]: 5.9%; partial response [PR]: 25.5%) in a heavily pretreated, PD-1-refractory population (median of 3 prior systemic therapies). Approximately 79.3% of patients experienced tumor burden reduction, and responses were durable: the median duration of response was 36.5 months, with 31.3% of responders (15/48) completing the 5-year assessment with ongoing responses. Four patients converted from PR to CR more than 1 year after infusion, and the longest response remained ongoing at 58.7 months. For overall survival, the median OS was 13.9 months, with a 5-year OS rate of 19.7% (nearly 1 in 5 patients alive at 5 years) [58].

Toxicities are substantial but largely confined to the peri treatment period, driven primarily by lymphodepleting chemotherapy and high-dose IL-2 (cytopenias, infections, capillary leak), and generally resolve within weeks. No new long-term safety signals emerged on extended follow-up.

Current challenges include logistical complexity, the requirement for adequate resectable tumor tissue, the need for specialized centers, and uncertainties regarding optimal sequencing with checkpoint inhibitors. These issues are being actively explored in ongoing studies of lifileucel in combination with pembrolizumab in the first-line setting (e.g., IOV-COM-202, TILVANCE-301), as well as in broader analyses of the evolving TIL-therapy landscape that emphasize careful patient selection and health-system readiness for wider implementation [53,54,55,56].

### 5.5. CNS-Directed Immunotherapy

Brain metastases represent one of the most frequent and lethal patterns of dissemination in advanced melanoma, affecting an estimated 40–50% of patients with stage IV disease and up to 30–40% at the time of metastatic diagnosis. Historically, survival after the detection of melanoma brain metastases was measured in weeks to a few months, but CNS-directed immunotherapy has dramatically altered this outlook. Combination immune checkpoint blockade with nivolumab plus ipilimumab is now a standard first-line approach for asymptomatic melanoma brain metastases, with long-term follow-up from the Australian ABC (Anti-PD-1 Brain Collaboration) trial showing approximately 48–51% overall survival at seven years, a result that has reframed brain involvement from uniformly terminal to potentially curable in a meaningful subset of patients [59,60].

Contemporary reviews highlight that intracranial responses and survival with immune checkpoint inhibitors in brain-metastatic melanoma can now approximate outcomes seen in extracranial disease for selected patients, particularly when CNS-directed immunotherapy is integrated early in the treatment course. At the same time, stereotactic radiosurgery (with or without whole-brain radiotherapy) and, for *BRAF*-mutant melanoma, CNS-penetrant BRAF/MEK inhibition remain essential components of a multimodal CNS-directed strategy, providing both rapid debulking and enhanced immunogenic cell death within the brain. Detailed profiling of melanoma brain metastases has highlighted the roles of microglia, astrocytes, and brain-resident endothelial and immune cells in creating an immunosuppressive niche, suggesting that effective CNS-directed therapy must reprogram these stromal compartments as much as it targets melanoma cells themselves. Preclinical studies suggest that inhibiting microglial RelA/NF-κB signaling can shift microglia from a pro-tumor to an anti-tumor phenotype, enhancing cytotoxic T-cell and NK-cell-mediated clearance of melanoma brain metastases and improving responses to checkpoint blockade [61]. Parallel work identifying focal adhesion kinase (FAK) as a driver of melanoma brain colonization has led to combination approaches in which FAK inhibitors are paired with other systemic agents to prevent or delay CNS spread in preclinical models, further emphasizing prevention as a key therapeutic goal. These advances support a view of melanoma brain metastases not only as a sanctuary site for resistant disease but also as a tractable therapeutic ecosystem—one in which CNS-tailored immunotherapy, radiation, targeted agents, and microenvironment-modulating approaches are deliberately co-designed to achieve durable intracranial control [62,63,64,65].

### 5.6. Resistance to Immunotherapy and Immune Evasion Mechanisms

Despite the transformative impact of checkpoint inhibitors, 40–65% of melanoma patients treated with PD-1 blockade exhibit resistance, either as primary resistance (no initial clinical response) or acquired resistance (an initial response followed by disease progression). As a result, unraveling the molecular and immunological mechanisms underlying ICI resistance has become a major research priority. Comprehensive genetic and transcriptomic profiling of tumors that progress on anti-PD-1 or anti-CTLA-4 therapy has revealed distinct resistance-associated signatures, highlighting the substantial heterogeneity of immune-escape mechanisms that enable tumors to evade anti-tumor immunity [44,66]. Tumors resistant to anti-PD-1 therapy typically display low levels of tumor-infiltrating lymphocytes, an immune-excluded phenotype marked by upregulation of MYC and cell-cycle-related genes, and reduced frequencies of PD-1+ CD8+ T cells. In contrast, tumors resistant to anti-CTLA-4 therapy generally retain immune infiltration but exhibit expanded populations of Foxp3+ regulatory T cells and preserved expression of immune-related gene programs. These distinct microenvironmental states point to divergent mechanisms of resistance, suggesting that different therapeutic strategies may be required to overcome them [44]. Genetic alterations linked to ICI resistance include loss-of-function mutations in key immune-regulatory genes (*NF1*, *TP53*, *PTEN*, *ARID1A*), defects in antigen presenting machinery (B2M, HLA class I), and oncogenic mutations that suppress anti-tumor immunity (*BRAF*, *NRAS*, *PIK3CA*). *BRAF*-mutant melanomas exhibit distinct patterns of ICI response compared to *BRAF* wild-type tumors, with transcriptomic analyses revealing enhanced immune-cell signatures in anti-PD-1-resistant *BRAF*-mutant tumors [67].

Molecular mechanisms of resistance include upregulation of alternative immune checkpoints such as LAG-3, TIM-3, and TIGIT; increased production of immunosuppressive cytokines including IL-10, TGF-β, expansion of suppressive myeloid populations such as myeloid-derived suppressor cells and tumor-associated macrophages; and metabolic reprogramming within the tumor microenvironment that restricts T-cell function. Notably, VEGF-A upregulation has emerged as a key contributor to anti-PD-1 resistance, with preclinical studies demonstrating that combining VEGF inhibition with PD-1 blockade can restore anti-tumor immunity and enhance therapeutic efficacy [66,68].

### 5.7. Immunotherapy Toxicity—Immune-Related Adverse Events

Although immunotherapy has dramatically improved survival outcomes, it is accompanied by a distinct toxicity profile known as immune-related adverse events (irAEs). These toxicities arise from dysregulated immune activation, where reinvigorated T cells and disrupted immune homeostasis led to inappropriate targeting of healthy tissues. Common irAEs include colitis/diarrhea (5–10%), pneumonitis (2–5%), hepatitis (3–5%), a range of endocrinopathies (thyroiditis, hypophysitis, type 1 diabetes), and various dermatologic toxicities. Especially, vitiligo—typically chronic—occurs more frequently in melanoma patients receiving ICI (specifically anti-PD-1 therapy) than in other cancer types and associated with enhanced anti-tumor responses [69,70,71,72].

Grade 3–4 immune-related adverse events (irAEs) occur in approximately 10–30% of patients receiving anti-PD-1 monotherapy and in up to 55% of those treated with the ipilimumab–nivolumab combination. Severe toxicities—including myocarditis (rare but potentially fatal), pneumonitis, colitis, and hepatitis—require prompt intervention with systemic corticosteroids and, in some cases, additional immunosuppressive agents. According to ASCO guidelines, management of grade 3 toxicities involves initiating high-dose corticosteroids (1–2 mg/kg/day methylprednisolone or equivalent), with escalation to agents such as infliximab or mycophenolate mofetil for steroid-refractory cases [69,71]. The use of corticosteroids, particularly at high doses, has been associated with diminished immune checkpoint inhibitor (ICI) efficacy, with clinical data in melanoma demonstrating reduced survival in patients receiving high-dose glucocorticoids for ipilimumab-induced hypophysitis [73].

A significant and often underappreciated challenge is the emergence of late onset irAEs, defined as toxicities occurring ≥3 months after final dose of ICI therapy. These late-onset irAEs encompass sarcoidosis-like reactions, arthritis, colitis, papillitis, and even neuropsychiatric manifestations. The mechanisms driving this delayed toxicity remain poorly understood but may involve epitope spreading, accumulation of autoreactive T cells, and long-lasting disruptions in Treg homeostasis. These observations highlight the importance of long-term clinical surveillance and heightened awareness of delayed toxicity optimize patient outcomes [70].

## 6. Combination Strategies

### 6.1. Rationale for Combining Targeted and Immunotherapy

As outlined in Section 4.2, BRAF/MEK inhibition initially increases melanoma antigen expression and CD8^+^ T-cell infiltration [42,74]. Table 2 provides a concise overview of currently used combination checkpoint inhibitor therapies targeting PD L1, PD 1, CTLA 4, LAG 3, and TIM 3, along with their mechanisms of action.

Concurrently, paradoxical MAPK pathway activation and recruitment of tumor-associated macrophages foster an immunosuppressive tumor microenvironment. Coupled with evidence that immune checkpoint inhibitors can overcome MAPK inhibitor resistance, these findings support combining targeted therapy with immune checkpoint blockade.

Triplet therapy combining the anti-PD-L1 antibody atezolizumab, BRAF inhibitor vemurafenib, and MEK inhibitor cobimetinib demonstrated superior efficacy over BRAF/MEK inhibition alone. In a phase III trial, progression-free survival was extended to 15.1 months versus 10.6 months (HR 0.78; *p* = 0.025), although interim analysis did not reveal a statistically significant overall survival benefit.

This leverages complementary mechanisms: targeted therapy achieves rapid tumor debulking, whereas checkpoint blockade sustains anti-tumor immunity. However, heightened toxicity and treatment complexity limit triplet therapy to carefully selected patients, particularly those with high tumor burden or rapidly progressive disease [43].

In melanoma, sequential and triple combination strategies aim to balance rapid disease control with durable response. Triple regimens that combine BRAF/MEK inhibition with anti-PD-1/PD-L1 therapy have shown biologic rationale and some clinical activity, but benefits in first-line trials have been inconsistent and toxicity a concern. More recently, investigational checkpoint-based triple regime, including PD-1/LAG-3/TIM-3 blockade, have entered early phase clinical testing as a strategy to overcome resistance in advanced melanoma; these remain experimental rather than standard of care.

### 6.2. Synergistic Effects and Clinical Trial Evidence

Sequential neoadjuvant immunotherapy with response-adapted adjuvant management is emerging as an effective strategy in stage III melanoma, as demonstrated in the phase II PRADO trial. Ninety-nine patients with clinically node-positive disease received two cycles of ipilimumab (1 mg/kg) plus nivolumab (3 mg/kg), yielding a 72% pathologic response rate, including 61% major pathologic response (MPR; ≤10% viable tumor). MPR was associated with favorable outcomes, with 24-month relapse-free and distant metastasis-free survival rates of 93% and 98%, respectively [28,29]. Multiple phase III trials are ongoing to validate checkpoint inhibitor-based approaches across melanoma subgroups (Table 3).

PRADO further demonstrated that MPR can guide surgical de-escalation. Therapeutic lymph node dissection was omitted in 59 of 60 patients with MPR, reducing morbidity and improving quality of life without compromising disease control. Survival outcomes remained high at 24 months (RFS ~93%, DMFS 98–100%), with manageable toxicity (22% grade 3–4 immune-related adverse events). In contrast, patients without MPR underwent lymph node dissection followed by risk-adapted adjuvant therapy (e.g., BRAF/MEK inhibition or nivolumab ± radiotherapy). These findings support pathologic response as a clinically actionable biomarker to tailor both surgical and adjuvant treatment intensity [28,29,75].

### 6.3. Intralesional, Infusional and Other Locoregional Therapies

#### 6.3.1. T-VEC Oncolytic Virus

Oncolytic viruses (OVs) are replication-competent viruses engineered or selected to preferentially infect, replicate within, and lyse malignant cells while sparing normal tissues. Talimogene laherparepvec (T-VEC) is the first-in-class OV to achieve regulatory approval and the only oncolytic virus approved by the US Food and Drug Administration (FDA) for melanoma, establishing proof-of-concept for this modality in solid tumors [76,77].

T-VEC oncolytic virus engineered with deletions in ICP34.5 and ICP47 and insertion of a GM-CSF transgene, enabling tumor-selective replication, direct oncolysis, and in situ vaccination through GM-CSF-mediated recruitment and activation of antigen-presenting cells. Administered by intralesional injection into all accessible melanoma lesions on a step-up dosing schedule, T-VEC received FDA approval in 2015 for unresectable cutaneous, subcutaneous, and nodal melanoma lesions recurrent after surgery, making it the first oncolytic virus licensed for cancer therapy. In the phase III OPTiM trial, T-VEC significantly improved durable response rate compared with GM-CSF (19% vs. 1–2%) and achieved an objective response rate of approximately 30%, including complete responses in about 17% of patients. The greatest benefit was observed in stage IIIB-IVM1a disease, and the therapy demonstrated a favorable tolerability profile dominated by low-grade flu-like symptoms and injection-site reactions [78,79,80,81].

Biologically, T-VEC exemplifies the dual oncolytic and immunomodulatory functions of OVs, inducing immunogenic tumor cell death, release of tumor antigens, and generating systemic T-cell-mediated responses capable of targeting uninfected lesions. Early phase studies combining T-VEC with PD-1 blockade (e.g., pembrolizumab) reported high response and complete response rates; however, the large phase III MASTERKEY-265 study (MASTERKEY trials are clinical studies investigating the combination of the oncolytic virus T-VEC and pembrolizumab) did not demonstrate a statistically significant improvement in progression-free or overall survival over pembrolizumab alone, and the combination has not gained regulatory approval. As a result, T-VEC currently occupies a niche as a locoregional immunotherapy for patients with predominantly injectable melanoma lesions. Ongoing efforts now focus on optimizing its integration with checkpoint inhibitors and surgical strategies, as well as applying insights from T-VEC to guide the development of next-generation oncolytic platforms [82].

#### 6.3.2. IL-2 Therapies

High-dose interleukin-2 (HD IL-2) represents a historically important milestone in melanoma immunotherapy. Approved by the FDA in 1998, it produced an overall response rate of approximately 16%, including 6% complete responses, with a small subset of patients achieving durable remissions lasting more than a decade However, high-dose IL-2 has largely been replaced by immune checkpoint inhibitors in the first-line setting because of its severe toxicity profile, including capillary leak syndrome, profound hypotension, and cardiac arrhythmias requiring intensive monitoring, as well as its modest response rate and substantial toxicity among non-responders [83,84].

Importantly, IL-2 should not be discussed solely as a systemic cytokine. For patients with unresectable stage III disease with satellite/in-transit metastases and selected stage IV M1a cutaneous/subcutaneous metastases, intralesional IL-2 (aldesleukin/Proleukin) has been incorporated into guideline-based locoregional management and a clinically relevant option when surgery is not feasible or when a lesion-directed approach is preferred. In a phase 2 melanoma series, intratumoral IL-2 produced complete local responses in 69% of evaluable patients, with only grade 1–2 toxicity reported, underscoring its favorable safety profile relative to systemic HD IL-2 [85]. This makes intralesional IL-2 particularly relevant for patients with limited-volume locoregional disease, older patients, or those with comorbidities who may not tolerate systemic cytokine therapy but could still benefit from a locoregional immunotherapy approach [86,87,88,89,90].

#### 6.3.3. Other Locoregional Therapies

Other established locoregional options for unresectable in-transit or cutaneous melanoma metastases include isolated limb perfusion (ILP) or isolated limb infusion (ILI) with melphalan with or without tumor necrosis factor-α (TNF-α), electrochemotherapy with bleomycin, intralesional rose bengal (PV-10), and topical imiquimod. Hyperthermic ILP/ILI permits delivery of very high doses of melphalan to a limb under tourniquet control, often with mild hyperthermia to enhance cytotoxicity, achieving overall response rates of approximately 50–75% and durable limb preservation in selected patients with limb-confined in-transit metastases, albeit with procedure-related morbidity and limited availability to high-volume centers. Addition of TNF-α to melphalan may increase regional tumor necrosis in some series but has not consistently improved outcomes over melphalan alone and is not universally available, particularly in North America. These regional chemotherapy approaches are increasingly being integrated with, or sequenced around, modern systemic immunotherapies, with ongoing trials exploring peri-procedural checkpoint blockade [91,92,93,94,95].

Electrochemotherapy combines brief high-voltage electric pulses with intratumoral or intravenous bleomycin to transiently increase cell membrane permeability, producing high local response rates and durable control of cutaneous and in-transit melanoma metastases with an acceptable toxicity profile. Intralesional PV-10 (10% rose bengal) is a small-molecule ablative immunotherapy that has demonstrated significant local and regional disease control, including regression of non-injected bystander lesions, in phase I/II studies of stage III–IV melanoma, with phase III and combination trials with pembrolizumab ongoing. Topical 5% imiquimod, a Toll-like receptor 7 agonist, has been used off-label for LM and cutaneous melanoma metastases, where case series and small studies report immune activation and partial or complete regressions, including when combined with modalities such as brachytherapy or other intralesional agents, and occasional reports describe regression of deeper metastases when applied to overlying skin. These locoregional strategies provide important options for patients with anatomically limited but otherwise unresectable disease, offering limb preservation, symptomatic relief, and potential immunologic synergy with systemic therapies [91,93,96,97].

Recent interest has also focused on developing engineered IL-2 variants that bias signaling toward effector CD8+ T cells while limiting regulatory T-cell expansion, thereby improving efficacy and reducing toxicity. Nemvaleukin alfa (ALKS 4230), an IL-2/CD25 fusion protein, has demonstrated pharmacodynamic activity and manageable safety in advanced melanoma, with clinical evaluation continuing both as monotherapy and in combination with pembrolizumab. Early studies combining IL-2-pathway agents with PD-1 inhibitors have shown encouraging activity, although clinical development remains active rather than definitive [83,84].

## 7. Adjuvant and Neoadjuvant Therapy

### 7.1. Stage II and III Disease: Sentinel Node Assessment and Risk Stratification

The role of SLNB in high-risk stage II melanoma is being actively debated around adjuvant immunotherapy. One argument for omission is that patients with pT3b–T4 tumors may be eligible for adjuvant anti-PD-1 therapy regardless of nodal status. However, this approach requires caution. Trials informing current practice, including adjuvant pembrolizumab (KEYNOTE-716, all trials using pembrolizumab, has been named as KEYNOTE) and adjuvant nivolumab (CheckMate 76K, all trials using nivolumab, has been named as CheckMate) enrolled only patients who underwent SLNB, limiting the ability to extrapolate their findings to SLNB-omitted populations. Beyond trial eligibility, SLNB continues to provide important prognostic information and may contribute to regional disease control and melanoma-specific survival, outcomes not fully replicated by systemic therapy alone [23,24,25,98,99,100].

Accordingly, although current NCCN guidelines allow for omission of SLNB in selected high-risk stage II patients after informed discussion, its role as both a staging and potentially therapeutic procedure is unresolved rather than definitively de-escalated [23,24,25,98,99,100]. Importantly, both trials demonstrated recurrence-free survival benefit but not overall survival benefit to date, further limiting justification for SLNB omission.

### 7.2. Adjuvant Immunotherapy: Pembrolizumab and Nivolumab

The KEYNOTE-716 trial demonstrated that adjuvant pembrolizumab significantly improved recurrence-free survival in patients with high-risk stage IIB/C melanoma compared with placebo [101]. Similarly, CheckMate 76K showed that adjuvant nivolumab provided a meaningful recurrence-free survival benefit over placebo in the same population [102]. These trials established the rationale for offering anti-PD-1 monotherapy to patients with high-risk stage II melanoma—marking the first clear evidence that adjuvant immunotherapy provides clinical benefit in a disease stage previously considered below threshold for systemic adjuvant treatment [25].

Adjuvant immunotherapy for stage III melanoma has been part of standard care for nearly a decade. Ipilimumab was the first agent approved in this setting based on EORTC 18071 trial, which demonstrated a significant improvement in recurrence-free survival compared with placebo (target HR 0.75; observed HR around 0.76 at longer follow-up) in patients with completely resected stage III disease. However, this benefit came at the cost of substantial toxicity: adverse events led to discontinuation in 245 of 471 patients (52%) who started ipilimumab, including 39% who discontinued during the initial four-dose induction period, and five patients (1%) died due to drug-related adverse events [103,104].

Subsequently, anti-PD-1 antibodies pembrolizumab and nivolumab demonstrated superior efficacy with a far more favorable safety profile, KEYNOTE-054 (EORTC 1325) trial showed that adjuvant pembrolizumab improved recurrence-free survival compared with placebo (HR about 0.56–0.57). Likewise, CheckMate 238 demonstrated that adjuvant nivolumab improved recurrence-free and distant metastasis-free survival compared with ipilimumab, while producing lower rates of grade 3–4 toxicity [105]. These trials established anti-PD-1 therapy—rather than ipilimumab—as the preferred adjuvant approach for resected stage III melanoma (and, for nivolumab, stage IIIB-C and IV disease) offering durable disease control with significantly reduced toxicity compared with high-dose CTLA-4 blockade [25,101,102,104].

Most recently, the combination of relatlimab plus nivolumab was approved for first-line unresectable or metastatic melanoma based on RELATIVITY-047 (HR 0.75, *p* = 0.0055); its role in the adjuvant setting is under active investigation [46]. The standard duration of adjuvant therapy is 12 months for both anti-PD-1 monotherapy and combination regimens. The key checkpoint blockade therapies approved by the FDA, Health Canada, and the EMA are summarized in Table 1. Although long-term overall survival data from these trials are still maturing, recurrence-free survival is a validated surrogate endpoint, supporting the clinical benefit for these adjuvant strategies [25].

### 7.3. Targeted Therapy in the Adjuvant Setting

Adjuvant BRAF/MEK inhibition is an established treatment option for patients with completely resected stage III *BRAF* V600-mutant melanoma. The combination of dabrafenib plus trametinib received FDA approval based on the phase III COMBI-AD trial (COMBInation with Trametinib versus two placebos in the ADjuvant treatment), which demonstrated a significant improvement in relapse-free survival compared with placebo [106]. Ongoing phase III studies—such as COLUMBUS-AD, evaluating encorafenib plus binimetinib in resected stage IIB/IIC *BRAF*-mutant melanoma—may further expand adjuvant targeted therapy options. In contrast, adjuvant vemurafenib monotherapy has not shown a meaningful clinical benefit [107]. When selecting adjuvant therapy for *BRAF*-mutant melanoma, clinicians must balance the toxicity profile and risk of acquired resistance with BRAF/MEK inhibition against the durable efficacy and distinct spectrum of immune-related adverse events seen with adjuvant anti-PD-1 immunotherapy [25].

### 7.4. Clinical Trial Design and Comparative Effectiveness

Direct comparative data between immunotherapy and targeted therapy have been limited. The phase III DREAMseq (Doublet, Randomized Evaluation in Advanced Melanoma Sequencing) trial demonstrated that first-line immunotherapy (nivolumab plus ipilimumab) followed by targeted therapy at progression improved overall survival compared with the reverse sequence in *BRAF* V600-mutant advanced melanoma. These findings support immunotherapy-first sequencing for most patients, although individualized approaches remain important for those with rapidly progressive disease or specific clinical constraints [108,109].

Important gaps remain, including long-term outcomes, quality of life, and cost-effectiveness. De-escalation strategies are also under investigation, particularly for patients achieving pathologic complete response after neoadjuvant therapy. The PRADO trial provides early support for response-adapted approaches, but randomized data are needed to define optimal treatment duration and ensure long-term safety [29].

## 8. Biomarkers, Liquid Biopsy and Precision-Medicine Implementation

Circulating tumor cells (CTCs) and circulating tumor DNA (ctDNA) represent tumor-derived cells and nucleic acids released into the peripheral circulation, providing a minimally invasive means of molecular assessment without the need for tissue biopsy. CTCs can be isolated from peripheral blood using immunomagnetic enrichment techniques targeting melanoma-associated markers such as MCAM, MCSP, ABCB5, and CD271. Higher CTC counts correlate with more advanced disease and poorer prognosis, with elevated levels observed in metastatic compared with primary melanoma [110].

ctDNA has emerged as a particularly promising biomarker for early detection, disease monitoring, and recurrence prediction in melanoma. Detection of *BRAF*-mutant ctDNA identifies patients at increased risk of relapse and may help guide decisions regarding adjuvant therapy intensification. However, significant challenges remain—most notably, the need for highly specific assays capable of distinguishing malignant signals from background variants, as benign nevi can shed detectable alleles into circulation. As a result, the current clinical use of liquid biopsy is focused primarily on treatment monitoring in established metastatic disease rather than population-level screening or early detection in asymptomatic individuals [110].

Prospective clinical validation of emerging biomarkers—including ctDNA dynamics, gene expression profiling (GEP), and multi-omics signatures demonstrating improved outcomes over current standard-of-care approaches—remains an urgent research priority [111]. Real-world evidence comparing comprehensive molecular profiling with traditional clinical judgment will be essential to determine whether the added cost and complexity of advanced biomarker testing translate into meaningful improvements in patient outcomes. Equally important is the standardization of biomarker methodologies, interpretation criteria, and reporting formats across institutions and laboratories. Consistent frameworks are critical for integrating biomarker-driven strategies into routine clinical practice. Guidelines from major oncology societies should be regularly updated to reflect emerging evidence and to support the safe, effective, and equitable implementation of biomarker-guided therapy.

Beyond ctDNA and circulating tumor cells, additional circulating biomarkers-such as cell-free RNA and serum proteins-are being explored for melanoma detection and prediction. Among these, S100B is one of the most extensively studied protein biomarkers; its serum levels correlate with disease stage and clinical outcomes, and elevated concentrations are associated with poorer survival in metastatic melanoma. However, its clinical utility is limited by low specificity, as S100B can also be elevated in other malignancies and benign conditions [110,112]. Pre-analytical variability in ctDNA assays stems from collection, processing, and extraction steps that differentially affect digital droplet PCR (ddPCR) and next-generation sequencing (NGS). Delayed plasma separation causes leukocyte lysis, diluting ctDNA with wild-type cfDNA and disproportionately impairing NGS’s detection of low-frequency variants due to its reliance on sufficient mutant allele fraction. NGS requires maximal cfDNA yield for library preparation; low input causes quantity-not-sufficient errors or poor coverage, whereas ddPCR’s partition-based absolute quantification is more robust to input variations. NGS library preparation introduces bias through fragmentation and adapter ligation efficiency dependent on cfDNA fragment size (~167 bp for ctDNA), while ddPCR amplifies predefined targets without library construction. Both assays suffer from hemolysis and improper storage, but NGS’s genome-wide scope amplifies pre-analytical noise across many loci, whereas ddPCR’s focused targeting limits this impact [113]. MicroRNA-based biomarkers further expand the liquid biopsy landscape, demonstrating the ability to distinguish melanoma from benign nevi and to predict progression-free survival independently of serum LDH. In parallel, extracellular vesicles, including exosomes carrying proteins and non-coding RNAs, are emerging as promising tools for melanoma detection and disease monitoring [110,112].

## 9. Special Populations and Disparities in Melanoma

Despite these advances, significant disparities persist in melanoma outcomes across racial and ethnic groups. Although melanoma represents only ~1% of skin cancers in African American populations (incidence: 1.0 per 100,000 vs. 33.9 per 100,000 in non-Hispanic Whites), it accounts for a disproportionate burden of skin cancer mortality. Black patients have a 5-year survival rate of 66–70%, compared to 90–92% for non-Hispanic White patients, with mortality rates approximately three-fold higher. This disparity is driven primarily by later-stage diagnosis: 55% of Black patients present with localized disease versus 86% of White patients, while 20% present with distant metastases compared to only 4% of Whites [114,115].

Acral and mucosal melanomas, which occur 4.1 times more frequently in Native American/Alaskan Native populations and are the most common subtypes in African Americans and Asians, are associated with worse prognoses and remain underrepresented in clinical trials. These subtypes have distinct molecular landscapes with lower *BRAF*-mutation rates (10–20% vs. 50% in cutaneous melanoma) and higher KIT mutation frequencies, and patients experience minimal long-term clinical benefit from immunotherapy, with median progression-free survival of only ~5.7 months. Addressing these disparities will require improved biological understanding of subtype-specific mechanisms, targeted efforts to overcome structural barriers to care, and more inclusive clinical research with intentional recruitment of underrepresented populations [114,115,116].

Long-term survivorship considerations are increasingly critical as more patients achieve durable remission. In one cohort, 14.7% of patients hospitalized with immune-related adverse events (irAEs) present 6–12 months after immune checkpoint inhibitor (ICI) initiation, and 10.8% present >12 months after starting therapy. The LATENT study defined late-onset irAEs as occurring ≥3 months after treatment completion and found nearly 15% of patients developed late toxicities, including cutaneous, gastrointestinal, hepatic, and rheumatologic events, with up to 2% presenting ultra-late-onset toxicities ≥ 12 months post-treatment. Long-term metastatic melanoma survivors treated with ipilimumab (median 5.6 years post-treatment) show 41% neurocognitive impairment, persistent fatigue (41%), and significantly worse physical, cognitive, and social functioning compared to matched controls. Developing and prospectively validating comprehensive survivorship care guidelines—including surveillance for late toxicities, recurrence monitoring, and second malignancy screening—will be essential to support this growing patient population [117,118,119,120].

Finally, rare melanoma subtypes and special populations present ongoing clinical challenges. Pediatric melanoma is biologically distinct from adult disease, with age-specific molecular features (higher *BRAF* V600E in adolescents but lower mutation burdens in younger children), yet optimal treatment strategies remain poorly defined due to limited trial data. Similarly, melanoma in immunocompromised patients—including those with HIV/AIDS or post-transplant immunosuppression—follows a more aggressive course: HIV-positive patients have significantly shorter disease-free (*p* = 0.03) and overall survival (*p* = 0.045), while solid organ transplant recipients have a 2.1- to 8-fold higher melanoma risk and poorer survival outcomes. These groups remain consistently underrepresented in clinical studies, underscoring the need for dedicated research to establish evidence-based management approaches [121,122].

## 10. Unanswered Questions in Melanoma Treatment

Despite transformative advances, the optimal sequencing of systemic therapies in *BRAF* V600-mutant metastatic melanoma remains unresolved. Data from DREAMseq and SECOMBIT now support an immunotherapy-first strategy followed by BRAF/MEK inhibition at progression for most patients [123]. However, DREAMseq excluded patients with brain metastases, rapidly progressive disease requiring urgent cytoreduction, and those with contraindications to dual checkpoint blockade, so upfront BRAF/MEK inhibition remains appropriate in selected high-risk cases. How to personalize sequencing decisions according to disease kinetics, metastatic burden, and patient-specific risk factors, and whether concurrent combination strategies offer additional benefit, remain active areas of investigation.

In resectable high-risk melanoma, the integration of neoadjuvant and adjuvant strategies is still being refined. The 2024 NADINA (Neoadjuvant Ipilimumab plus Nivolumab versus Standard Adjuvant Nivolumab in Macroscopic Stage III Melanoma) trial established neoadjuvant nivolumab plus ipilimumab as superior to adjuvant-only therapy, with approximately 12-month event-free survival of ~84% versus ~57% and nearly 60% of patients achieving a major pathologic response and omitting adjuvant therapy. Nevertheless, important questions remain regarding the optimal neoadjuvant regimen, the ideal number of preoperative doses, and how best to adapt adjuvant therapy according to pathologic response. ctDNA and radiomic signatures are promising tools for refining response-adapted strategies, but they require prospective validation [124,125].

A deeper understanding of response and resistance to immunotherapy is another major unmet need. Durable responses in some patients contrast sharply with primary resistance or rapid relapse in others, even among tumors with similar clinicopathologic features. Tumor-intrinsic mechanisms, such as antigen presentation defects and oncogenic signaling alterations, interact with tumor-extrinsic factors, including T-cell infiltration, myeloid cell composition, and stromal influences, but their relative contributions remain incompletely defined [126].

Reliable predictive biomarkers remain an additional unmet need. Although PD-L1 expression and tumor mutational burden correlate with response in some settings, their clinical utility is limited by insufficient sensitivity and specificity. Integrative multi-omics approaches that combine genomic, transcriptomic, spatial immune profiling, and ctDNA dynamics appear more promising, but they still require standardization and prospective validation [126,127].

A related challenge is response assessment, particularly pseudo progression, in which radiologic tumor enlargement reflects immune-cell infiltration rather than true disease progression. Although iRECIST provides a useful framework, distinguishing immune-related change from true progression remains difficult in practice. ctDNA monitoring has shown promise as a complementary tool for this purpose, with reported high accuracy for distinguishing pseudo progression from true progression. More robust diagnostic frameworks that integrate imaging, circulating biomarkers, and clinical context are needed to avoid premature discontinuation of effective therapy [127,128,129].

### 10.1. Treatment Sequencing

Given these uncertainties, the most practical approach is to stratify treatment decisions according to resectability, *BRAF* V600 mutation status, performance status, and early biological response rather than to search for a single universally optimal regimen. In resectable stage III or oligometastatic melanoma, neoadjuvant immunotherapy is increasingly favored in fit patients, supported by improved event-free survival with perioperative pembrolizumab in SWOG S1801 and high pathological response rates with neoadjuvant ipilimumab plus nivolumab in high-risk disease. In unresectable stage III or IV melanoma, first-line therapy should be guided by both molecular status and disease kinetics: patients with *BRAF* V600-mutant melanoma and rapidly progressive, symptomatic, or organ-threatening disease may benefit from upfront targeted therapy, whereas clinically stable patients are generally better served with initial immunotherapy, consistent with DREAMseq, which showed superior survival when nivolumab plus ipilimumab preceded dabrafenib plus trametinib [108,127,130,131,132,133].

A practical sequencing framework follows this risk-adapted approach. In resectable high-risk stage III or IV disease in fit patients, neoadjuvant PD-1-based therapy followed by surgery and response-adapted adjuvant treatment informed by pathologic findings and ctDNA dynamics is increasingly supported. In unresectable or metastatic *BRAF* wild-type melanoma, checkpoint blockade is generally preferred as first-line therapy, with nivolumab plus ipilimumab reserved for patients requiring maximal long-term disease control and nivolumab plus relatlimab considered when minimizing toxicity is a priority. In unresectable or metastatic *BRAF* V600-mutant disease, immunotherapy remains appropriate for most fit patients; however, BRAF/MEK inhibition may be favored when rapid disease control is required, with early reassessment and a low threshold to switch therapy if ctDNA rises, radiographic response is lacking, or the clinical condition worsens. In frail patients or those with poor performance status, simplified approaches are usually warranted, most often single-agent PD-1 therapy or targeted therapy in symptomatic *BRAF*-mutant cases. Across all scenarios, patient preference remains central because treatment choices increasingly involve trade-offs among response kinetics, durability of benefit, toxicity risk, and treatment burden. Although ctDNA negativity after neoadjuvant or early systemic therapy is a promising signal for treatment de-escalation, prospective validation is still required before response-adapted discontinuation strategies can be used routinely [133,134,135,136,137].

### 10.2. Controversies

The role of sentinel lymph node biopsy (SLNB) in melanoma is increasingly selective rather than obsolete. While effective adjuvant immunotherapies have reduced the immediate therapeutic impact of nodal staging in some patients, current evidence still supports SLNB as an important tool for staging and prognostication. A more evidence-based approach is not broad omission, but selective consideration in well-defined low-risk cohorts, while preserving SLNB for patients whose nodal status informs eligibility for neoadjuvant or adjuvant systemic therapy [108,137,138,139,140].

The trade-off between durability of response and treatment-related toxicity is especially clear when comparing nivolumab plus ipilimumab with PD-1/LAG-3 blockade. Extended follow-up from RELATIVITY-047 shows sustained benefit of nivolumab plus relatlimab over nivolumab monotherapy, with improved response and progression-free survival and a more favorable toxicity profile than historical ipilimumab-containing regimens. Even so, nivolumab plus ipilimumab remains the most established option for deep and durable remissions in selected patients, albeit with substantially higher rates of grade 3–4 immune-related adverse events and treatment discontinuation. In practice, the central question is therefore not which regimen is universally superior, but which option best fits the patient’s disease kinetics, tolerance for immune toxicity, and need for rapid, profound tumor control [47,134,135].

## 11. Conclusions

Over the past decade and a half, melanoma treatment has undergone a profound transformation driven by the development of targeted therapies and immune checkpoint inhibitors. These advances have substantially improved survival outcomes, with long-term survival now achievable in a significant proportion of patients with advanced disease.

The therapeutic landscape continues to evolve with the introduction of novel modalities, including tumor-infiltrating lymphocyte (TIL) therapy, engineered cytokines, oncolytic viruses, and next-generation targeted agents. These approaches expand the arsenal of available treatments and offer new opportunities to improve outcomes, particularly for patients who do not benefit from current standards of care.

A key direction for the field is the development of rational, biology-driven treatment strategies. Increasing emphasis is being placed on adaptive approaches that tailor therapy intensity based on individual response—allowing for de-escalation in patients with deep and durable responses and escalation for those with resistant disease. This shift reflects a broader move toward personalized treatment paradigms. Equally important is the need to balance efficacy with tolerability. As survival improves, long-term toxicity, quality of life, and patient preferences are becoming central considerations in treatment decision-making. Optimizing this balance will be essential to delivering meaningful clinical benefit.

Looking ahead, continued progress will depend on integrating advances in tumor biology, immunology, and clinical trial design. The refinement of combination strategies coupled with more precise patient stratification has the potential to further improve outcomes while minimizing unnecessary toxicity.

## Figures and Tables

**Table 1:** The key checkpoint blockade therapies for Melanoma approved in the US, Canada and Europe.

Therapy Class/Example	FDA	Health Canada	EMA/Europe
Anti-CTLA-4 (e.g., Ipilimumab)	Approved	Approved & reimbursed	Approved & widely reimbursed
Anti-PD-1 (e.g., pembrolizumab, nivolumab)	Approved (advanced/adjuvant)	Approved & reimbursed	Approved & widely reimbursed
Anti-CTLA-4 + PD-1 (ipilimumab + nivolumab)	Approved	Approved	Approved, reimbursed in many European countries.
LAG-3 + PD-1 (nivolumab-relatlimab, Opdualag)	Approved	Approved	Approved, limited reimbursement
BRAF/MEK inhibitors (e.g., dabrafenib-trametinib)	Approved	Approved & reimbursed	Approved, widely reimbursed across Europe.
TIL therapy (lifileucel, Amtagvi)	Approved 2024	Approved 2025	Application withdrawn *
Oncolytic virus (Imlygic/T-VEC)	Approved	Approved	Approved; reimbursement available in a subset of European countries.

* Lifileucel (Amtagvi) in Europe was withdrawn by the sponsor on 22 July 2025; this was a sponsor withdrawal, not a regulatory withdrawal or rejection by EMA.

**Table 2:** Overview of Mechanisms of Action for Checkpoint-Inhibitor Combinations Used in Melanoma Immunotherapy. This table summarizes anti-LAG-3 inhibitors alongside combination regimens targeting PD-1, PD-L1, CTLA-4, and TIM-3, outlining their primary ligands and key downstream signaling or metabolic effects.

Triple-Combo	Targeted Receptor/Axis	Main Ligand(s) in the Axis	Downstream Signaling in Melanoma
Anti-PD-1 + anti-CTLA-4 + anti-LAG-3	PD-1, CTLA-4, LAG-3 inhibitory receptors on activated/exhausted T cells (and Tregs for CTLA-4)	PD-1 ↔ PD-L1/PD-L2; CTLA-4 ↔ CD80/CD86; LAG-3 ↔ MHC-II (canonical) and FGL1, etc.	PD-1 engagement suppresses proximal TCR signaling and leads to reduced PI3K/AKT and Ras/MEK/ERK activity; blockade helps restore effector signaling and cytokine production CTLA-4 competes with CD28 for binding to CD80/CD86, thereby reducing co-stimulation and IL-2 production and T cell proliferation; CTLA-4 blockade enhances T cells priming and co-stimulation and may also diminish Treg-mediated suppression LAG-3 engagement at the immunological synapse inhibits TCR signaling in part through interference with Lck-coreceptor interactions mediated by its EP motif. LAG-3 blockade is intended to relieve inhibitory signaling during T cells activation.
Anti-PD-1 + anti-CTLA-4 + anti-TIM-3	PD-1, CTLA-4, TIM-3 inhibitory receptors (TIM-3 often up on dysfunctional/exhausted T cells)	PD-1 ↔ PD-L1/L2; CTLA-4 ↔ CD80/CD86; TIM-3 ↔ Galectin-9 and other ligands (such as CEACAM1, phosphatidylserine, HMGB1)	PD-1 signaling suppresses TCR-associated phosphorylation events and dampens downstream PI3K/AKT and Ras/MEK/ERK pathways; PD-1 blockade helps restore effector T-cell programs. CTLA-4 blockade restores CD28-mediated costimulatory signaling during T-cell priming, enhancing Il-2 production and proliferation of T cells. TIM-3 engages multiple ligands—including Gal-9, CEACAM1, PtdSer, HMGB1—to restrain T-cell effector function; TIM-3 blockade aims to reverse this inhibitory signaling in exhausted T cells within tumors.
Anti-PD-1 + anti-LAG-3 + anti-TIM-3	PD-1, LAG-3, TIM-3 inhibitory receptors on exhausted/dysfunctional TILs.	PD-1 ↔ PD-L1/PD-L2; LAG-3 ↔ MHC-II (and ligands such as FGL1); TIM-3 ↔ Gal-9, CEACAM1, phosphatidylserine, HMGB1.	PD-1 blockade releases suppression of TCR downstream pathways (often discussed as restoring PI3K/AKT and Ras/MEK/ERK activity and cytokine output). LAG-3 blockade relieves an additional, partly distinct TCR-inhibitory mechanism at the immunological synapse (including interference with Lck-co-receptor coupling). TIM-3 blockade targets a separate exhaustion-associated inhibitory module driven by multiple ligands including Gal-9, CEACAM1, PtdSer and HMGB1to further restore effector function.

The bidirectional arrow ↔ means: “binds to”.

**Table 3:** Summary of phase, population, treatment, primary endpoint, and status of selected recent or ongoing melanoma trials.

Trial Name	Phase	Target Population	Treatment	Primary Endpoint	Estimated Completion
HARMONY Head-to-Head (NCT06246916)	3	Stage III/IV unresectable or metastatic melanoma	Fianlimab + cemiplimab vs. relatlimab + nivolumab	ORR, PFS, OS	2031 (up to 72 months)
PRISM-MEL-301 (NCT06112314)	3	Stage III/IV unresectable or metastatic cutaneous melanoma	Brenetafusp + nivolumab vs. nivolumab ± relatlimab	PFS (BICR per RECIST 1.1)	2029 (up to 57 months)
SUPRAME (NCT06743126)	3	Previously treated unresectable or metastatic cutaneous melanoma	IMA203 (PRAME CAR-T) vs. investigator’s choice	PFS (BICR per RECIST 1.1)	2030 (up to 5 years)
NADINA (ongoing follow-up) NCT04949113	3	Macroscopic Stage III melanoma (resectable)	Neoadjuvant ipilimumab + nivolumab vs. adjuvant nivolumab	Event-free survival (EFS)	Ongoing (18-month EFS: 80.8% vs. 53.9%)
RELATIVITY-098 (terminated) NCT05002569	3	Resected Stage III/IV melanoma (adjuvant)	Nivolumab + relatlimab vs. nivolumab alone	RFS	Results published 2025
LNS8801-MEL NCT06624644	1/2	Unresectable treatment-refractory melanoma	LNS8801 (GPER agonist) ± pembrolizumab	Safety, ORR, PFS	Ongoing
IGNYTE-3 (NCT06264180)	3	Advanced melanoma after anti-PD-1 and anti-CTLA-4 failure	VO + nivolumab vs. standard treatment choice	OS, PFS, ORR	Ongoing
Neo IRENIE (NCT06999980)	2	Stage III cutaneous melanoma and mucosal melanoma	Neoadjuvant immunotherapy regimen	pCR, EFS, ORR, MRR, OS	Ongoing
Binimetinib and Imatinib for Unresectable Stage III-IV KIT-Mutant Melanoma	2	Unresectable stage III/IV KIT-mutant melanoma	Binimetinib + imatinib	ORR, AE, PFS, OS, CBR	Ongoing
Grand SLAM NCT06488482	3	High-risk resected melanoma (adjuvant)	6 months vs. 12 months nivolumab + relatlimab	RFS, DMFS at 2 years	Ongoing (1792 patients)
IL13Rα2 CAR-T (Stanford-led) NCT04119024	1	Advanced melanoma and solid tumors	IL13Rα2-targeting CAR-T cells	Safety, tolerability	Recruiting 2025–2026
ABC Trial (NCT02374242)	2	Stage IV melanoma with brain metastases	Ipilimumab + nivolumab vs. nivolumab alone	intracranial response rate (complete or partial response); assessed via modified RECIST criteria	ongoing for long-term survival follow-up
CheckMate 238 (NCT02388906)	3	Resected Stage IIIB-IIIC/IV melanoma	Nivolumab vs. ipilimumab	RFS, defined as time from randomization to recurrence or death	completed, with final 9-year outcomes reported

Abbreviations: PFS, progression-free survival; OS, overall survival; HR, hazard ratio; ORR, Objective response rate; RFS, relapse free survival; DMFS, Distant Metastasis-Free Survival.

## Data Availability

No new data were generated or analyzed in support of this research. This article is a review and does not report original data.

## Abbreviations

**Abbreviation**	**Full term**
AIOM	Associazione Italiana di Oncologia Medica (Italian Association of Medical Oncology)
AJCC	American Joint Committee on Cancer
ANZMTAG	Australia and New Zealand Melanoma Trials Alliance Guidelines Group
ASCO	American Society of Clinical Oncology
ASCO/SSO	American Society of Clinical Oncology/Society of Surgical Oncology
BRAF	v-Raf murine sarcoma viral oncogene homolog B1
CD8	Cluster of differentiation 8
CDKN2A	Cyclin-dependent kinase inhibitor 2A
cfDNA	Cell-free DNA
CLND	Completion lymph node dissection
CNS	Central nervous system
CR	Complete response
CTC	Circulating tumor cell
ctDNA	Circulating tumor DNA
CTLA-4	Cytotoxic T-lymphocyte-associated protein 4
DMFS	Distant metastasis-free survival
DTIC	Dacarbazine
EADO	European Association of Dermato-Oncology
EFS	Event-free survival
EMA	European Medicines Agency
EMEA	European Agency for the Evaluation of Medicinal Products
EORTC	European Organisation for Research and Treatment of Cancer
ESMO	European Society for Medical Oncology
FAK	Focal adhesion kinase
FDA	Food and Drug Administration
GEP	Gene expression profiling
GM-CSF	Granulocyte-macrophage colony-stimulating factor
HD IL-2	High-dose interleukin-2
HR	Hazard ratio
ICI	Immune checkpoint inhibitor
ICP	Immune checkpoint
IL-2	Interleukin-2
ILI	Isolated limb infusion
ILP	Isolated limb perfusion
irAEs	Immune-related adverse events
iRECIST	Immune-response evaluation criteria in solid tumors
LAG-3	Lymphocyte activation gene-3
LDH	Lactate dehydrogenase
LM	Lentigo maligna
MAPK	Mitogen-activated protein kinase
MHC	Major histocompatibility complex
MHC-I	Major histocompatibility complex class I
MHC-II	Major histocompatibility complex class II
MMP	Matrix metalloproteinase
MMS	Mohs micrographic surgery
MPR	Major pathologic response
MSLT-II	Multicenter Selective Lymphadenectomy Trial II
MSS	Melanoma-specific survival
MTD	Maximum tolerated dose
NCCN	National Comprehensive Cancer Network
NGS	Next-generation sequencing
NSCLC	Non-small cell lung cancer
ORR	Objective response rate
OS	Overall survival
OV	Oncolytic virus
pCR	Pathologic complete response
PD-1	Programmed cell death protein 1
PD-L1	Programmed death-ligand 1
PFS	Progression-free survival
PI3K	Phosphatidylinositol-3-kinase
PV-10	Rose bengal disodium (PV-10)
qPCR	Quantitative polymerase chain reaction
RFS	Recurrence-free survival
RTK	Receptor tyrosine kinase
SLNB	Sentinel lymph node biopsy
SSO	Society of Surgical Oncology
SWOG	Southwest Oncology Group
T-VEC	Talimogene laherparepvec
TCR	T-cell receptor
TIL	Tumor-infiltrating lymphocyte
TLND	Therapeutic lymph node dissection
TME	Tumor microenvironment
TMZ	Temozolomide
TNF-α	Tumor necrosis factor-alpha
WLE	Wide local excision
